# Biological Sex Disparities in the Economic Burden of Tobacco Use: A Comparative Analysis between Men and Women in China

**DOI:** 10.3390/ijerph21080980

**Published:** 2024-07-26

**Authors:** Rong Zheng, Lingyun Meng, Aduqinfu He

**Affiliations:** School of International Trade and Economics, University of International Business and Economics, No. 10, Huixin Dongjie, Chaoyang District, Beijing 100029, China; 202200152004@uibe.edu.cn (L.M.); adqf819@outlook.com (A.H.)

**Keywords:** biological sex disparities, tobacco use, economic costs, China

## Abstract

To examine the impact of tobacco use on the economic costs between biological sex differences, we utilized propensity score matching and human capital methods to analyze the economic costs associated with smoking. Our findings reveal a nuanced pattern in the economic burden: although men who smoke bear a higher overall economic cost, the individual impact on women who smoke is significantly more profound. As a result, there exists a distinct disparity in the distribution of economic consequences stemming from tobacco use between men and women.

## 1. Introduction

Generally, men tend to use all tobacco products at higher rates than women. In 2020, 22.3% of the world’s population used tobacco, with 36.7% among men and 7.8% among women [1]. Such disparities may stem from a combination of physiological, cultural, and behavioral factors. In China, this biological sex gap in smoking is particularly stark, with men smoking at a rate of 47.6% compared to 1.9% for women [2], where men smoking is often associated with notions of masculinity or social status in the region, and women smoking is stigmatized or frowned upon.

A substantial body of evidence illustrates the harmful effects of smoking on health, including impaired lung function [3], accelerated atherosclerosis [4], premature aging [5], and an increased risk of lung cancer [6]. The chronic nature of these health issues leads to a continual rise in healthcare costs, impacting not just patients and their families but also society as a whole. Additionally, evidence shows differences between men and women in tobacco use and how these distinctions could contribute to various diseases [7,8]. This disparity may disproportionately affect financial security based on biological sex due to the differences in earnings and labor opportunities between men and women.

The objective of this study is to examine the biological sex-based differences in the economic burden attributable to smoking in China. Understanding these variations is pivotal for crafting effective tobacco control policies.

## 2. Data and Methods

We utilized data from China to scrutinize the economic ramifications of tobacco use among men and women spanning the years 2014 to 2018. For descriptive statistics and detailed variable definitions related to our data, see Supplementary Tables S1–S3. It is important to note that in this study, smoking refers specifically to commercial cigarette smoking. In the context of China, “tobacco” generally refers to cigarettes, as the use of smokeless tobacco is minimal and electronic cigarettes were not included in our survey questions related to smoking. Additionally, in this study, the definition of individuals who smoke includes those who smoke occasionally [9]. According to the World Health Organization, these costs can be categorized into direct and indirect costs [10]. Direct costs denote the monetary value of goods and services attributable to tobacco-induced diseases, while indirect costs encompass the economic toll stemming from diminished labor productivity due to illness or premature mortality among people who smoke. Adhering to the World Health Organization’s methodology, we employed propensity score matching (PSM) to compute the direct economic costs. Additionally, we integrated the human capital approach with disability-adjusted life years (DALYs) to evaluate the indirect economic costs.

Direct cost computations drew upon China Family Panel Survey (CFPS) data in conjunction with National Bureau of Statistics of China (NBSC) data. By utilizing propensity score matching (PSM) to identify individuals in the control group who closely matched the personal characteristics of those in the treatment group, we measured the difference in costs between the two groups to estimate the direct economic costs of smoking for each individual. The CFPS data furnished individualized medical outlays, encompassing both inpatient and outpatient expenses. Additionally, to mitigate the influence of extreme values, we winsorized the annual total health expenditure (top 1%). The treatment group consisted of individuals with a history of smoking. The analyses in this paper show that the proportion of self-rated health among people who smoke is higher than that among people who do not smoke, suggesting that the smoking population may be more likely to disregard their health. Using people who smoke as the treatment group might lead to self-selection bias.

Acknowledging the delayed manifestation of tobacco-related health hazards and the primary reason why people who smoke consider quitting is their concern for personal health [11], we further categorized this group into people who are currently smoking and people who have previously smoked but are now abstaining. To ensure precise matching outcomes, we incorporated 17 covariates, spanning personal demographics, socioeconomic status, and health metrics. Following meticulous data cleansing protocols, the average annual sample size for PSM analysis was approximately 18,600 for women and 18,500 for men.

To ascertain the total direct economic costs of tobacco use in China, we applied the following formula:

Total Direct Economic Costs = Direct Economic Costs × Proportion of Tobacco Users in the Total Population × Total Population(1)

This computation encapsulates the financial impact directly attributed to tobacco use within the population.

For the calculation of indirect costs, we drew upon the methodology proposed by Sung et al. [12] and Long et al. [13], which integrates disability-adjusted life years (DALYs) with the human capital approach, utilizing GDP per capita as a measure of productivity. The formula employed for this calculation is the following:

Total Indirect Economic Costs = GDP Per Capita × Disability-Adjusted Life Years × Productivity Weight(2)

Here, a productivity weight of 0.5 is assigned to the overall population, acknowledging the variations in productivity among different demographic groups [14]. DALYs, a pivotal metric for assessing the years lost due to tobacco use, are predominantly sourced from the global burden of disease (GBD) study.

Utilizing the aforementioned formulas and data, we computed the indirect economic costs of tobacco use in China for the years 2014, 2016, and 2018.

Finally, by summing the direct costs and indirect costs, we derived the comprehensive total costs attributable to tobacco use, thereby providing a comprehensive perspective on the economic burden imposed by tobacco consumption in China.

## 3. Results

### 3.1. Direct Economic Cost

**Figure 1:**
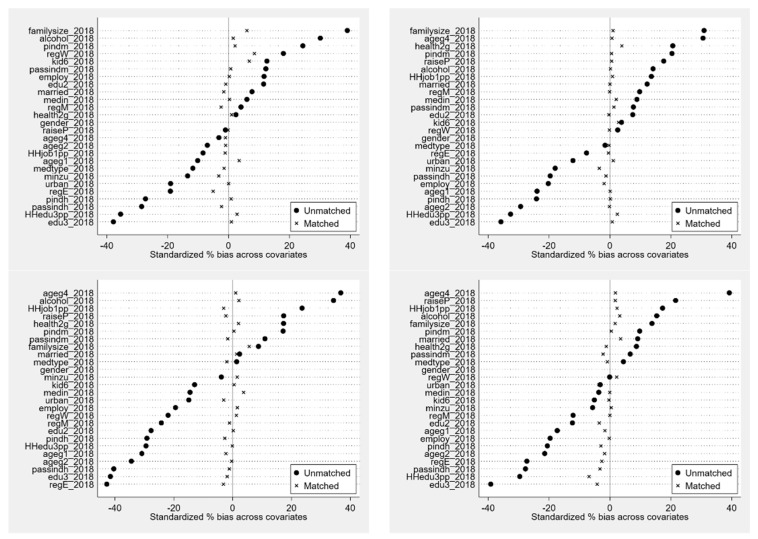
Standardized differences in PSM covariates. Note: the results of balance tests should not exceed 10%.

We employed kernel matching to align the control and treatment groups. Illustrating the process using data from 2018 (as depicted in Figure 1), we presented the results of balance tests conducted post-PSM. The figure delineates the top and bottom sections for men and women who smoke, while the left and right sides represent those who are currently smoking and those who have previously smoked, respectively. For a comprehensive view of the balance test results from 2014 to 2018, see Supplementary Figure S1.

Table 1 reveals an intriguing pattern: among people who are currently smoking, treatment effects fail to significantly influence direct costs across biological sexes, underscoring the insidious nature of tobacco use. Interestingly, within the group of individuals who have previously smoked, women show no substantial direct costs, while men exhibit notable increases in direct costs.

With fewer women who have previously smoked and negligible direct costs observed among them, men continue to shoulder the primary burden of smoking-related direct economic costs. In 2014, 2016, and 2018, the total direct economic cost attributed to men who have previously smoked amounted to RMB 75.07 billion, RMB 76.15 billion, and RMB 93.19 billion, respectively.

### 3.2. Indirect Economic Cost

Table 2 presents the calculated indirect costs derived from Formula (2). In the first column, the total indirect costs of tobacco use are displayed, revealing a stark biological sex disparity. Men shoulder the bulk of these indirect economic burdens, with costs approximately 4.6 times higher than those incurred by women. Notably, over the four years, both men and women experienced significant growth in indirect economic costs, with increases of 46.02% and 46.82%, respectively. Interestingly, the population of women exhibited slightly higher growth rates.

The last two columns of Table 2 illustrate the discrepancies in the indirect costs between active and passive smoking across men and women. Passive smoking specifically refers to any current exposure to secondhand tobacco smoke at home or work. It is important to note that only people who smoke non-daily are considered to be exposed to secondhand smoke [15,16].

In terms of the indirect costs stemming from active smoking, men who smoke bear notably higher economic burdens compared to their women counterparts. By 2018, the indirect costs for men who smoke had surged by 46.30% to reach RMB 1615.52 billion. In contrast, women who smoke experienced a 47.73% increase during the same period, with indirect costs amounting to RMB 213.99 billion—merely 13.25% of the expenses incurred by men who smoke. This disparity underscores the heavier economic toll of tobacco consumption on men, the primary users, while also highlighting a swifter rise in associated costs among women, despite their significantly lower expenditure.

While the indirect costs of passive smoking in China may seem comparatively modest, the repercussions remain concerning, particularly for women. In 2018, women incurred indirect costs amounting to approximately RMB 172.22 billion—a striking 1.25 times higher than the costs borne by men.

### 3.3. Total Economic Cost

**Figure 2:**
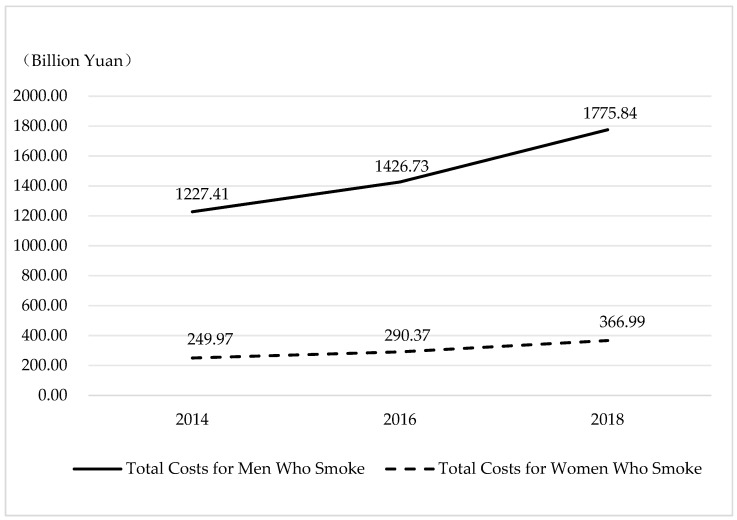
Total economic costs of tobacco use by different biological sexes.

Figure 2 depicts a consistent uptrend in the aggregated costs for tobacco users from 2014 to 2018 across both men and women. Specifically, the costs for men who smoke surged from RMB 1227.41 to 1775.84 billion—a notable 44.68% increase. Similarly, for women who smoke, the total economic costs escalated from RMB 249.97 to 366.99 billion, marking a 46.82% increase over the same period. This trend underscores the mounting economic ramifications of smoking, highlighting the imperative for interventions aimed at curbing tobacco-related expenditures and safeguarding public health.

Given the substantial difference between direct and indirect costs, and the negligible direct costs incurred by women who smoke, this study reveals that women who smoke bear indirect economic costs nearly three times greater than their men counterparts. Specifically, the average annual indirect cost for men who smoke stands at RMB 3210.10, while for women who smoke, it significantly surpasses this figure at RMB 9407.02—an increase of 50.32%, which outpaces the 45.89% rise observed among men who smoke. This marked contrast can be attributed, in part, to women’s heightened susceptibility to chronic obstructive pulmonary disease (COPD) and increased risk of biological sex-specific conditions such as cervical cancer, osteoporosis, and premature menopause.

The CFPS database, utilized in this study for calculating the direct costs, is a comprehensive, nationwide, interdisciplinary social tracking survey project. For computing the indirect costs, we employed the GBD database (global burden of disease), which stands as the most extensive and detailed scientific study on the global burden of diseases, injuries, and risk factors. Therefore, the findings of this study are applicable to the entirety of China.

## 4. Limitations

This study is subject to several limitations. First, the data limitations inherent in the China Family Panel Studies (CFPS) pose significant challenges to the precise quantification of the direct costs associated with secondhand smoke exposure. Second, the data are specifically limited to commercial cigarette smoking, indicating that our calculated direct costs represent a conservative estimation, with the actual figures likely exceeding this threshold. Third, our study is constrained by the inherent limitations of the global burden of diseases (GBD) study. Specifically, the availability of primary data and the reliability of results are dependent on the predictive validity of the models beyond the sample used in this study [17]. Finally, the use of a productivity weight of 0.5 in this study does not address potential discrepancies in the productivity weights between men and women, an issue that warrants further investigation in future research.

## 5. Conclusions

Our analysis reveals a distinct pattern of economic costs associated with smoking in China: while men, who constitute the majority of smokers, bear the bulk of economic costs associated with smoking, women, who smoke less frequently, encounter lower direct expenses from active smoking but disproportionately shoulder the repercussions of secondhand smoke exposure. At an individual level, women face heightened economic burdens linked to tobacco use, characterized by both larger magnitudes and growth rates compared to men. This disparity highlights the disproportionate financial strain that women endure due to tobacco, despite their lower smoking prevalence. These findings underscore the critical need for tobacco control policies that consider biological sex differences, aiming to address significant economic disparities in the impact of tobacco and promote equity in public health.

## Figures and Tables

**Table 1:** Direct economic costs of tobacco use by different biological sexes.

Year	People Who Have Previously Smoked				People Who Are Currently Smoking			
	Men		Women		Men		Women	
	ATT	t-Value	ATT	t-Value	ATT	t-Value	ATT	t-Value
2014	709.68 ***	4.38	297.12	0.87	−77.90	−0.63	−221.13	−0.99
	(161.89)		(343.45)		(122.86)		(223.97)	
2016	711.95 ***	3.67	721.24	1.84	−168.41	−1.07	−112.13	−0.36
	(194.14)		(392.63)		(157.52)		(314.57)	
2018	864.50 ***	3.68	597.11	1.46	−92.50	−0.50	−201.99	−0.55
	(234.94)		(408.71)		(185.84)		(367.04)	

Note: Average Treatment Effect on the Treated (ATT) is a fundamental concept in causal inference that quantifies the average impact of a treatment on the individuals who have actually undergone the treatment. Standard error in parentheses; *** *p* < 0.001; the radius is one-quarter of the standard error.

**Table 2:** Indirect economic costs of tobacco use by different biological sexes (billion RMB).

Year	Biological Sex	Total Indirect Costs	Smoking	Passive Smoking
2014	Men	1152.33	1104.28	96.72
	Women	249.97	144.85	118.42
2016	Men	1350.58	1294.55	112.95
	Women	290.37	168.52	137.09
2018	Men	1682.65	1615.52	137.81
	Women	366.99	213.99	172.22

Note: The calculation method results in a slight discrepancy where the sum of DALYs for active smoking and passive smoking exceeds the DALYs directly attributable to tobacco use.

## Data Availability

Not applicable.

## Supplementary Materials

The following supporting information can be downloaded at: https://www.mdpi.com/article/10.3390/ijerph21080980/s1, Figure S1: Standardized Differences in PSM Covariates (2014–2018); Table S1: Descriptive Statistic—Men; Table S2: Descriptive Statistic—Women; Table S3: Definitions of the variables.
