# Supplementary material for: Biological Sex Disparities in the Economic Burden of Tobacco Use: A Comparative Analysis between Men and Women in China
Source: Int J Environ Res Public Health. 2024 Jul 26;21(8):980. doi: 10.3390/ijerph21080980 (PMC11353992; doi:10.3390/ijerph21080980)
Supplement: Supplementary file 1 [file ijerph-21-00980-s001.zip › ijerph-3091580-supplementary.pdf]

FigureS1. Standardized Differences in PSM Covariates (2014-2018)

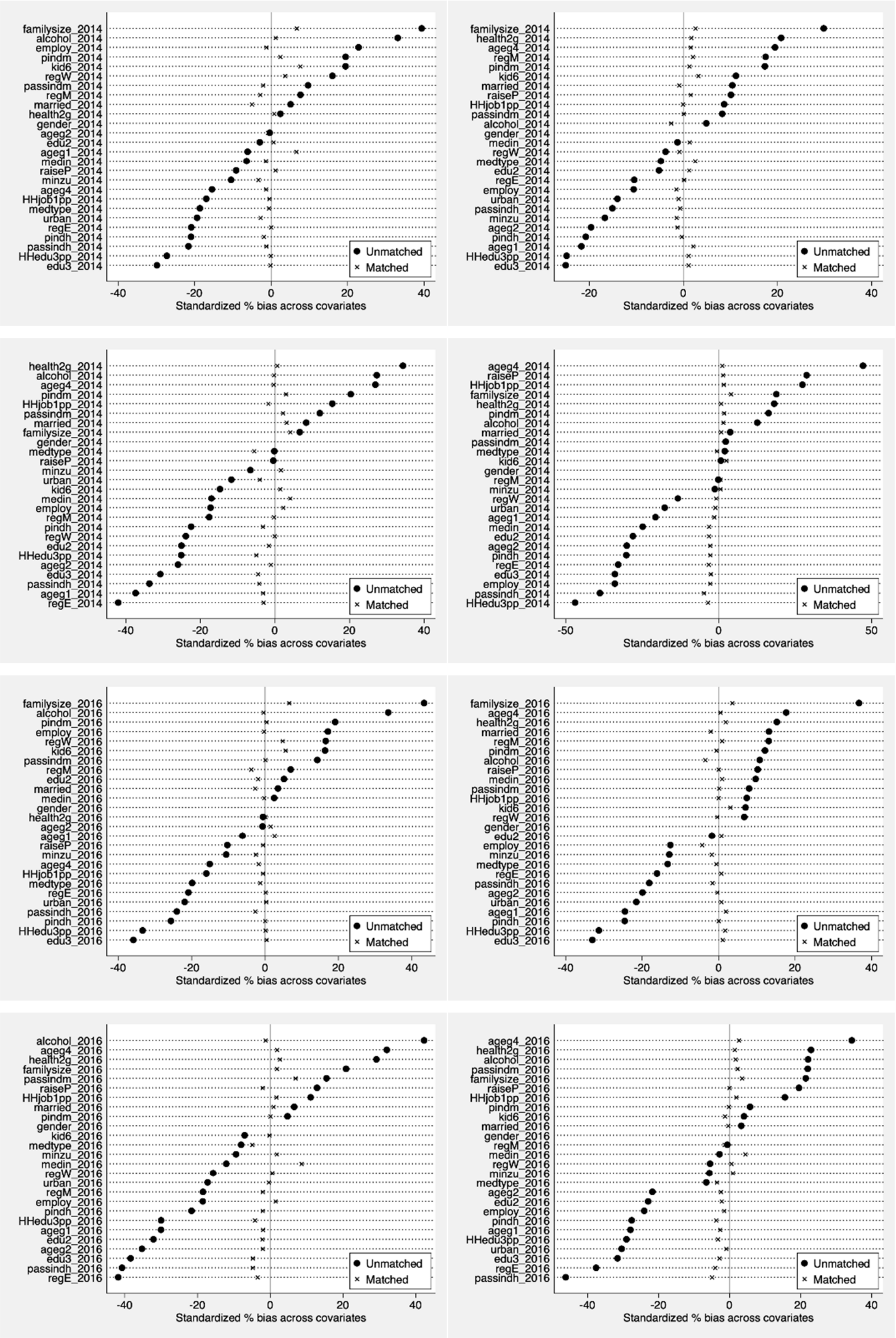

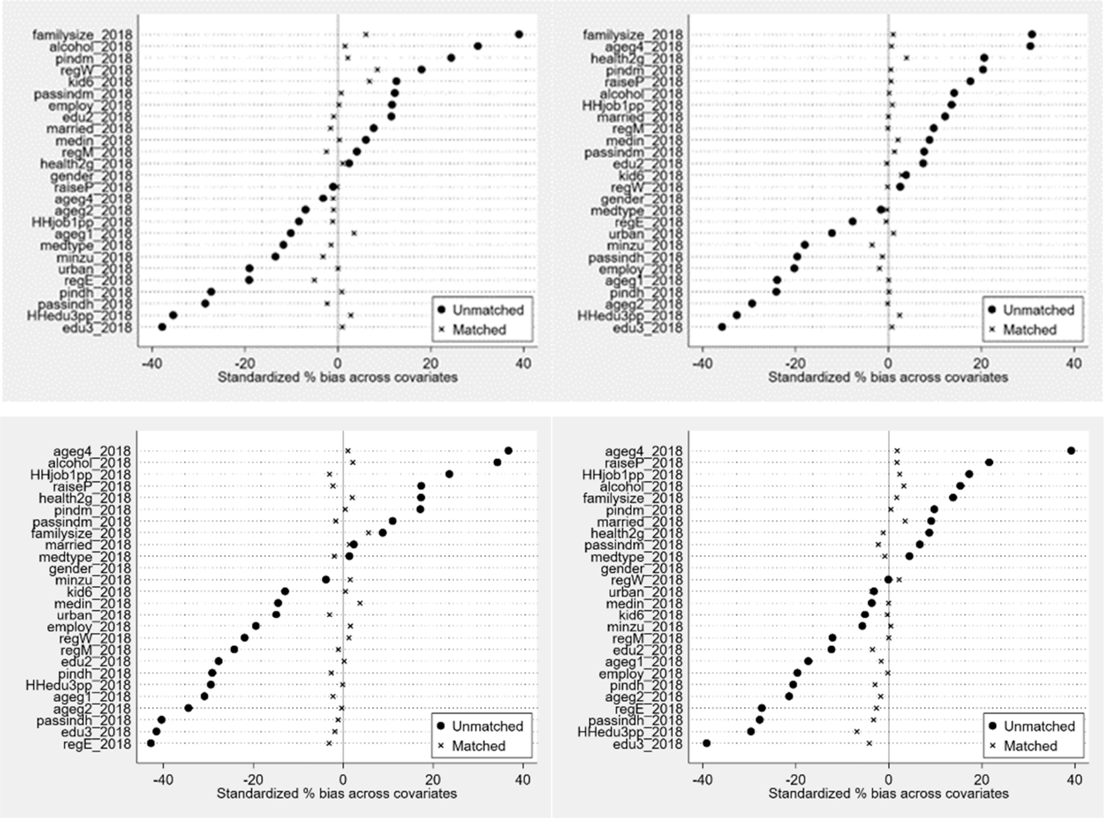

TableS1. Descriptive Statistic-Men

variable	2014		2016		2018	
	Mean	Std. Dev	Mean	Std. Dev	Mean	Std. Dev
Outcome variable						
Tmedicalcost	1534.09	4122.79	1810.27	4868.72	2157.20	5723.70
Treatment variable						
Curstreat	0.78	0.41	0.78	0.41	0.77	0.42
Forstreat	0.54	0.50	0.57	0.50	0.57	0.50
Covariate						
Ageg1	0.24	0.43	0.23	0.42	0.21	0.41
Ageg2	0.26	0.44	0.25	0.43	0.24	0.43
Ageg3	0.35	0.48	0.36	0.48	0.36	0.48
Ageg4	0.15	0.36	0.16	0.37	0.19	0.39
Nationality	0.08	0.27	0.08	0.28	0.09	0.28
Married	0.76	0.43	0.76	0.43	0.76	0.43
Employ	0.78	0.42	0.77	0.42	0.77	0.42
Urban	0.46	0.50	0.47	0.50	0.49	0.50
RegEN	0.13	0.34	0.13	0.33	0.12	0.32
RegE	0.32	0.47	0.33	0.47	0.34	0.47
RegM	0.25	0.43	0.24	0.43	0.23	0.42
RegW	0.30	0.46	0.31	0.46	0.31	0.46
Edu1	0.43	0.50	0.42	0.49	0.42	0.49
Edu2	0.48	0.50	0.47	0.50	0.47	0.50

Edu3	0.09	0.29	0.11	0.31	0.11	0.31
Pindl	0.20	0.40	0.20	0.40	0.19	0.40
Pindm	0.60	0.49	0.59	0.49	0.60	0.49
Pindh	0.21	0.40	0.21	0.41	0.21	0.41
Passindl	0.20	0.40	0.20	0.40	0.20	0.40
Passindm	0.60	0.49	0.60	0.49	0.60	0.49
Passindh	0.20	0.40	0.20	0.40	0.20	0.40
Medin	0.91	0.28	0.91	0.29	0.91	0.28
Familysize	4.31	1.99	4.31	2.05	4.24	2.06
HHjob1pp	0.24	0.27	0.23	0.27	0.22	0.27
HHedu3pp	0.07	0.17	0.08	0.18	0.08	0.19
Kid6	0.24	0.43	0.23	0.42	0.22	0.41
RaiseP	0.26	0.26	0.27	0.26	0.30	0.28
Alcohol	0.28	0.45	0.26	0.44	0.25	0.43
Health2g	0.26	0.44	0.29	0.45	0.25	0.43
Medtype	0.35	0.48	0.39	0.49	0.41	0.49

TableS2. Descriptive Statistic-Women

variable	2014		2016		2018	
	mean	Std. Dev	mean	Std. Dev	mean	Std. Dev
Outcome variable						
Tmedicalcost	1828.53	4045.13	2120.24	4699.61	2591.22	5583.81
Treatment variable						
Curstreat	0.10	0.30	0.10	0.30	0.09	0.29
Forstreat	0.07	0.26	0.08	0.27	0.09	0.28
Covariate						
Ageg1	0.24	0.43	0.24	0.42	0.21	0.41
Ageg2	0.24	0.43	0.23	0.42	0.23	0.42
Ageg3	0.36	0.48	0.36	0.48	0.36	0.48
Ageg4	0.16	0.36	0.17	0.38	0.20	0.40
Nationality	0.08	0.27	0.09	0.28	0.09	0.29
Married	0.77	0.42	0.77	0.42	0.78	0.41
Employ	0.62	0.48	0.61	0.49	0.62	0.49
Urban	0.48	0.50	0.49	0.50	0.50	0.50
RegEN	0.14	0.34	0.13	0.34	0.13	0.33
RegE	0.32	0.47	0.32	0.47	0.33	0.47
RegM	0.25	0.43	0.25	0.43	0.24	0.43
RegW	0.29	0.45	0.30	0.46	0.30	0.46
Edu1	0.54	0.50	0.53	0.50	0.52	0.50
Edu2	0.38	0.49	0.38	0.48	0.37	0.48
Edu3	0.08	0.26	0.09	0.29	0.10	0.30
Pindl	0.20	0.40	0.20	0.40	0.21	0.40
Pindm	0.60	0.49	0.61	0.49	0.60	0.49
Pindh	0.19	0.40	0.19	0.39	0.19	0.39
Passindl	0.20	0.40	0.20	0.40	0.20	0.40

Passindm	0.60	0.49	0.60	0.49	0.60	0.49
Passindh	0.20	0.40	0.20	0.40	0.20	0.40
Medin	0.90	0.29	0.91	0.29	0.91	0.28
Familysize	4.38	1.98	4.40	2.04	4.36	2.05
HHjob1pp	0.25	0.27	0.25	0.27	0.24	0.27
HHedu3pp	0.07	0.17	0.08	0.18	0.08	0.19
Kid6	0.26	0.44	0.25	0.44	0.24	0.43
RaiseP	0.28	0.26	0.28	0.26	0.32	0.27
Alcohol	0.03	0.17	0.03	0.16	0.03	0.17
Health2g	0.34	0.47	0.38	0.48	0.32	0.47
Medtype	0.36	0.48	0.40	0.49	0.42	0.49

TableS3. Definitions of the variables

Variables	Measure
**Dependent variables**	
Tmedicalcost	Total annual medical expenses, including hospitalization expenses and non-hospitalization expenses.
**Independent variables**	
Curstreat	0=Else 1=Current smoker
Forstreat	0=Else 1=Former smoker
**Control variables**	
Ageg1	0=Else 1=15–29
Ageg2	0=Else 1=30–44
Ageg3	0=Else 1=45–64
Ageg4	0=Else 1=⩾65
Gender	0 = Female 1 = Male
Nationality	0= Han (HA) 1= Else
Married	0=Single 1= Else
Employ	0=Unemployed 1=Employed
Urban	0= Else 1=Urban
RegEN	0= Else 1= Northeast region
RegE	0= Else 1=East region
RegM	0= Else 1= Midland
RegW	0= Else 1=West region
Edu1	0= Else 1=Illiterate and primary school
Edu2	0= Else 1=Junior high school and high school
Edu3	0= Else 1=College degree and above
Pindl	0=Else 1= < Quintile 1 (low income)
Pindm	0=Else 1= Quintile 1-4 (middle income)
Pindh	0=Else 1= > Quintile 4 (high income)
Passindl	0=Else 1= < Quintile 1 (low assets)
Passindm	0=Else 1= Quintile 1-4 (middle assets)
Passindh	0=Else 1= > Quintile 4 (high assets)
Medin	0=Else 1= Have insurance
Familysize	Total family population

HHjob1pp	Proportion of unemployed people in the family
HHedu3pp	Proportion of family members with college education or above to the total family population
Kid6	0=Else 1= household has children under 6 years old
RaiseP	Proportion of family population under 6 years old and over 64 years old to the total family population
Alcohol	0=Else 1= Drink more than 3 times a week.
Health2g	0= Unhealthy 1=Healthy
Medtype	0=Else 1= General or specialized hospital
